# Analysis of acute-phase toxicities of intensity-modulated proton therapy using a model-based approach in pharyngeal cancer patients

**DOI:** 10.1093/jrr/rraa130

**Published:** 2020-12-29

**Authors:** Koichi Yasuda, Hideki Minatogawa, Yasuhiro Dekura, Seishin Takao, Masaya Tamura, Nayuta Tsushima, Takayoshi Suzuki, Satoshi Kano, Takatsugu Mizumachi, Takashi Mori, Kentaro Nishioka, Motoyasu Shido, Norio Katoh, Hiroshi Taguchi, Noriyuki Fujima, Rikiya Onimaru, Isao Yokota, Keiji Kobashi, Shinichi Shimizu, Akihiro Homma, Hiroki Shirato, Hidefumi Aoyama

**Affiliations:** Department of Radiation Oncology, Hokkaido University Hospital, North-15 West-7, Sapporo, Japan; Department of Radiation Oncology, Hokkaido University Hospital, North-15 West-7, Sapporo, Japan; Department of Radiation Oncology, Faculty and Graduate School of Medicine, Hokkaido University, North-15 West-7, Sapporo, Japan; Department of Radiation Oncology, Hokkaido University Hospital, North-15 West-7, Sapporo, Japan; Department of Radiation Medical Science and Engineering, Faculty and Graduate School of Medicine,Hokkaido University, North-15 West-7, Sapporo, Japan; Department of Medical Physics, Hokkaido University Hospital, North-15 West-7, Sapporo, Japan; Department of Medical Physics, Hokkaido University Hospital, North-15 West-7, Sapporo, Japan; Department of Otolaryngology-Head and Neck Surgery, Faculty and Graduate School of Medicine,Hokkaido University, North-15 West-7, Sapporo, Japan; Department of Otolaryngology-Head and Neck Surgery, Faculty and Graduate School of Medicine,Hokkaido University, North-15 West-7, Sapporo, Japan; Department of Otolaryngology-Head and Neck Surgery, Faculty and Graduate School of Medicine,Hokkaido University, North-15 West-7, Sapporo, Japan; Department of Otolaryngology-Head and Neck Surgery, Faculty and Graduate School of Medicine,Hokkaido University, North-15 West-7, Sapporo, Japan; Department of Oral Radiology, Graduate School of Dental Medicine, Hokkaido University, Hokkaido University, North-13 West-7, Sapporo, Japan; Department of Radiation Medical Science and Engineering, Faculty and Graduate School of Medicine,Hokkaido University, North-15 West-7, Sapporo, Japan; Department of Radiation Oncology, Hokkaido University Hospital, North-15 West-7, Sapporo, Japan; Department of Radiation Oncology, Faculty and Graduate School of Medicine, Hokkaido University, North-15 West-7, Sapporo, Japan; Department of Radiation Oncology, Hokkaido University Hospital, North-15 West-7, Sapporo, Japan; Department of Radiology, Boston Medical Center, Boston University School of Medicine, Boston, MA, USA; Department of Radiation Oncology, Faculty and Graduate School of Medicine, Hokkaido University, North-15 West-7, Sapporo, Japan; Department of Biostatistics, Faculty and Graduate School of Medicine, Hokkaido University, North-15 West-7, Sapporo, Japan; Department of Medical Physics, Hokkaido University Hospital, North-15 West-7, Sapporo, Japan; Department of Radiation Medical Science and Engineering, Faculty and Graduate School of Medicine,Hokkaido University, North-15 West-7, Sapporo, Japan; Department of Radiation Medical Science and Engineering, Faculty and Graduate School of Medicine,Hokkaido University, North-15 West-7, Sapporo, Japan; Department of Otolaryngology-Head and Neck Surgery, Faculty and Graduate School of Medicine,Hokkaido University, North-15 West-7, Sapporo, Japan; Department of Radiation Oncology, Faculty and Graduate School of Medicine, Hokkaido University, North-15 West-7, Sapporo, Japan; Department of Radiation Oncology, Faculty and Graduate School of Medicine, Hokkaido University, North-15 West-7, Sapporo, Japan

**Keywords:** IMPT, pharyngeal cancer, robust optimization

## Abstract

Pharyngeal cancer patients treated with intensity-modulated proton therapy (IMPT) using a model-based approach were retrospectively reviewed, and acute toxicities were analyzed. From June 2016 to March 2019, 15 pharyngeal (7 naso-, 5 oro- and 3 hypo-pharyngeal) cancer patients received IMPT with robust optimization. Simulation plans for IMPT and intensity-modulated X-ray therapy (IMXT) were generated before treatment. We also reviewed 127 pharyngeal cancer patients with IMXT in the same treatment period. In the simulation planning comparison, all of the normal-tissue complication probability values for dysphagia, dysgeusia, tube-feeding dependence and xerostomia were lower for IMPT than for IMXT in the 15 patients. After completing IMPT, 13 patients completed the evaluation, and 12 of these patients had a complete response. The proportions of patients who experienced grade 2 or worse acute toxicities in the IMPT and IMXT cohorts were 21.4 and 56.5% for dysphagia (*P* < 0.05), 46.7 and 76.3% for dysgeusia (*P* < 0.05), 73.3 and 62.8% for xerostomia (*P* = 0.43), 73.3 and 90.6% for mucositis (*P* = 0.08) and 66.7 and 76.4% for dermatitis (*P* = 0.42), respectively. Multivariate analysis revealed that IMPT was independently associated with a lower rate of grade 2 or worse dysphagia and dysgeusia. After propensity score matching, 12 pairs of IMPT and IMXT patients were selected. Dysphagia was also statistically lower in IMPT than in IMXT (*P* < 0.05). IMPT using a model-based approach may have clinical benefits for acute dysphagia.

## INTRODUCTION

Radiation therapy (RT) is the mainstay treatment for head and neck cancer. Although intensity-modulated radiation therapy (IMRT) reduces the risk of xerostomia compared to 3-D conformal radiation therapy (3D-CRT) [1], patients still experienced various types of toxicities with IMRT, and these toxicities compromised quality of life during and after RT [2]. In locally advanced nasopharyngeal, oropharyngeal and hypopharyngeal cancer patients, whole neck irradiation from the retropharyngeal to supraclavicular region is needed, leading to severe toxicities.

In proton beam therapy, most of the energy is deposited at a specific depth in the path (the Bragg peak). This unique characteristic allows proton therapy to deliver a better dose distribution than photon-based RT [3]. In recent years, scanning methods have been developed that allow wide-range treatment that is adequate for whole-neck irradiation [4]. The technology of intensity-modulated proton therapy (IMPT), in which all spots or lines of protons from all fields are simultaneously optimized by using an inverse optimization method and the delivery of highly conformal and homogeneous dose, has also been developed [5]. Planning simulation studies suggested the superiority of IMPT for reducing toxicities compared to IMRT using X-ray (IMXT) for pharyngeal patients [4, 5].

Robust optimization methods for IMPT planning have recently been developed that account for the setup and range uncertainties [6, 7]. Although these techniques are considered useful for conducting accurate and precise IMPT over the course of treatment, clinical reports of IMPT with robust optimization are limited. To our knowledge, no clinical studies have reported or compared the acute toxicities of IMPT with IMXT during the same treatment period including all pharyngeal cancer patients. The present study investigated whether pharyngeal cancer patients treated with IMPT had significantly fewer acute toxicities than IMXT-treated patients.

## MATERIALS AND METHODS

### IMPT patients

From June 2016 to March 2019, 7 nasopharyngeal, 5 oropharyngeal and 3 hypopharyngeal cancer patients received IMPT at our institution. The first patient was 18 years old and received proton therapy in the category of pediatric cancer in the Japanese health care system, which was approved by the Ministry of Health, Labor and Welfare (MHLW) in April 2016. The other 14 patients were in the advanced medical care system and were approved by the MHLW and were registered as an observational study in the national case registration (UMIN000022917). Our institutional review board approved this protocol for retrospective analysis in June 2019 (No. 019-0017). Patient characteristics are summarized in Table 1.

**Table 1:** Patient characteristics

**Characteristics**	**IMPT cohort**		**IMXT cohort**		***P* value**
	Number	%	Number	%	
	15		127		
Sex					0.7865
Male	14	93.3	116	91.3	
Female	1	6.7	11	8.7	
Age (years)					0.7616
18–65	9	60.0	71	55.9	
>65	6	40.0	56	44.1	
Tumor classification^a^					0.9906
T 0–2	9	60.0	76	59.8	
T 3–4	6	40.0	51	40.2	
Node classification^a^					0.1989
N 0–1	6	40.0	73	57.5	
N 2–3	9	60.0	54	42.5	
Primary site					0.0073^*^
Nasopharynx	7	46.7	15	11.8	
Oropharynx	5	33.3	58	45.7	
Hypopharynx	3	20.0	54	42.5	
Treatment field of radiation therapy					
With prophylactic bilateral neck irradiation	15	100.0	125	98.4	0.7985
With prophylactic ipsilateral neck irradiation	0	0	1	0.8	
Without prophylactic neck irradiation (=local field)	0	0	1	0.8	
Dose and fractions of radiation therapy					0.0054^*^
70 Gy (E) in 35 fractions	13	86.7	121	95.3	
71 Gy (E) in 33 fractions	0	0.0	6	4.7	
71.4 Gy (E) in 34 fractions	2	13.3	0	0.0	
Treatment modalities					0.0724
CCRT	10	66.7	104	81.9	
IC followed by CCRT	2	13.3	19	15.0	
CCRT followed by adjuvant Cx	3	20.0	4	3.1	
Toxicities before treatment					NA
Dysphagia ≥Grade 2	0/15	0.0	0/125	0.0	
Dysgeusia ≥Grade 2	0/15	0.0	0/118	0.0	
Xerostomia ≥Grade 2	0/15	0.0	0/116	0.0	

^a^According to the UICC TNM-classification, 8th edition.

^*^Asterisks indicate statistical significance of difference at p<0.05.

Gy (E) = gray (cobalt gray equivalent), Cx = chemotherapy, NA = not applicable.

### IMPT planning with robust optimization

VQA (Hitachi, Ltd., Hitachi, Japan) was used for IMPT planning. We usually used 3 fields, which included one anterior oblique (75 or 285 degrees) and two posterior oblique (160 and 200 degree angles) fields. Relative biological effectiveness (RBE) for proton beam therapy was estimated to be 1.1. In IMPT planning, 99% of the clinical target volume (CTV) received the prescribed dose. Dose constraints for organs at risk (OARs) are summarized in Supplementary Table 1, see online supplementary material. Set-up uncertainties of 3 mm and range uncertainties of 3.5% of the beams’ nominal ranges were assumed in the robust optimization [8]. For evaluation, we generated six plans in which the isocenter was shifted 3 mm in six directions (anterior, posterior, left, right, superior and inferior). The criterion was that a 98% volume of the CTV received at least 95% of the prescribed dose in all six scenarios [9].

### The dose delivery approach

The schema of adaptive IMPT and the dose delivery approach is shown in Supplementary Fig. 1, see online supplementary material. Two plans were used for the entire treatment [10]. The first plan was generated using the first computed tomography (CT) scan. Basically, 46 gray equivalents (GyE) was prescribed to CTV1, which included low-risk area, prophylactic neck lymph node volume, and high-risk area, microscopic tumor infiltration volume around primary tumor and metastatic lymph nodes. The basic policy for selection of the prophylactic neck node level is shown in Supplementary Table 2, see online supplementary material. At the third week of the treatment, a second CT was conducted, and a second plan was generated using the second CT. A dose of 24 GyE was prescribed to the high-risk area, and the total dose was 70 GyE in 35 fractions. However, the daily dose was changed to 2.1 GyE, and the total dose was changed to 71.4 GyE in 34 fractions in two cases. The PROBEAT-RT system (Hitachi. Ltd, Hitachi, Japan) was used for the delivery of IMPT [11].

### Comparison of IMPT and IMXT planning simulations

A Pinnacle^3^ V90 (Philips, Fitchburg, WI, USA) was used for IMXT planning. To generate the planning target volume (PTV), a 3-mm margin was added to the CTV. The prescribed dose was received by 95% of the PTV. The dose constraints were the same as in IMPT. Seven static fields (40, 70, 150, 180, 220, 290 and 320 degree angles) were used for IMXT using step-and-shoot methods. These plans were basically generated by the Pinnacle^3^ auto-planning module [12] with the same parameters that were used in the clinical setting to compensate for the quality of the plans. All plans were approved by a board-certificated radiation oncologist who had 10 or more years of experience with head and neck RT.

In the planning comparison, we checked the mean dose of OARs and calculated the normal-tissue complication probability (NTCP) for toxicities such as dysphagia [13], dysgeusia [14], tube-feeding dependence [15] and xerostomia [16]. The plans were compared by the cancer board, who decided which plan to implement.

### IMPT treatment and evaluation

All 15 patients received concurrent chemoradiotherapy (CCRT) with cisplatin. The chemotherapy regimens are shown in Supplementary Table 3, see online supplementary material. Efficacies and toxicities were evaluated using the Response Evaluation Criteria in Solid Tumors (RECIST) v1.1 and the Common Terminology Criteria for Adverse Events (CTCAE) v4.0 guidelines, respectively. Acute toxicities were evaluated as the worst grade of toxicities during the RT or up to 1 month after RT. In a patient with a prophylactic percutaneous endoscopic gastrostomy (PEG) feeding tube, the grade of dysphagia was evaluated by the symptom and alteration of eating and swallowing, not the use of PEG.

### IMXT cohort

To evaluate the acute toxicities of IMPT, we also reviewed nasopharyngeal, oropharyngeal and hypopharyngeal cancer patients with IMXT and the same dose-delivery method as that for IMPT during the same treatment period. The definitions of CTVs were also the same for IMXT and IMPT during this period. A total of 127 patients received definitive IMXT with concurrent chemotherapy. Their characteristics are summarized in Table 1. The chemotherapy regimens are shown in Supplementary Table 3. We calculated the cumulative dose of cisplatin in both cohorts. The averages of the cumulative doses in the IMPT and IMXT cohorts were 300 and 274 mg, respectively, and the difference was not statistically significant (*P* = 0.1382). Their acute toxicities were retrospectively evaluated.

### Effects of anatomical changes on the IMPT plan with robust optimization

The first CT scan, the regions of interests from the first plan (first ROIs) and the second CT scan were imported into deformable image registration (DIR) software MIM-MAESTRO (MIM Software, Cleveland, OH, USA). The first ROIs were deformed to fit the second CT scan. After import of the deformed first ROIs and second CT scan to VQA, the first plan was re-calculated on the second CT scan without re-optimization. Dose to target and OARs in the first plan on the first CT scan and re-calculated second CT scan were evaluated through a dose–volume histogram (DVH) analysis.

### Statistical analysis

Chi-square tests were used to compare categorical variables and the rate of acute toxicities between the IMPT and IMXT cohorts. In the DVH comparison, the target conformity and homogeneity index [17] were evaluated using the same PTV (volume added 3 mm to the CTV) and 70 or 71.4 GyE were prescribed in both the IMPT and IMXT plans. The continuous variables in the DVH and NTCP were compared using Wilcoxon rank sum tests. The Wilcoxon signed rank test was used for paired continuous variables in the DVH. Candidate variables that were initially entered for multivariate analysis for acute toxicities were the type of RT (IMPT or IMXT), prescription dose (as a continuous variable), sex (male or female), age (≤65 or >65 years), disease site (nasopharynx, oropharynx or hypopharynx), T stage (T1–2 or T3–4), N stage (N0–1 or N2–3), stage (1–2 or 3–4) and chemotherapy [CCRT only, induction chemotherapy (IC) + CCRT or CCRT + adjuvant chemotherapy]. The variables were selected using forward stepwise selection with the lowest Bayes information criterion. Multivariate analyses were performed with logistic regression analysis using the selected variables. Matched-pair analysis was performed for the independent factors generated from multivariate analysis. Each IMPT patient was matched to an IMXT patient based on the factors used for multivariate analysis. A caliper width of 0.2 of the standard deviation of the logit of the propensity score was used for patient selection. All *P*-values were 2-sided, and *P* < 0.05 was considered statistically significant. Analyses were performed with JMP Pro v14.0.0 (SAS Institute Inc., Cary, NC, USA).

## RESULTS

### Planning comparison for IMPT candidates

**Fig. 1.**
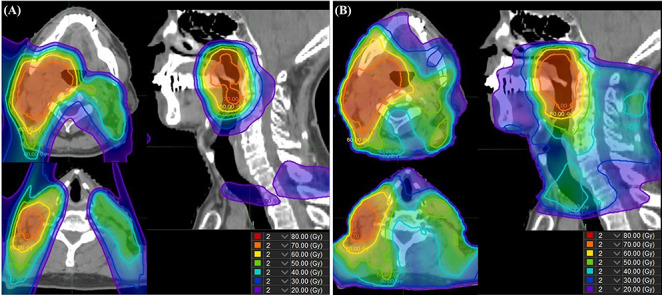
Case presentation of the planning comparison. IMPT (**A**) and IMXT (**B**) planning simulation in an oropharyngeal cancer patient. The IMPT plan had a lower dose to the oral cavity and larynx than the IMXT plan.

The results of the planning comparison are summarized in Table 2. The conformity was better in IMPT than in IMXT, and the homogeneity was comparable between the two plans. The dose to OARs and the NTCP value were significantly lower in IMPT than in IMXT (all *P* < 0.05). Delta NTCP (NTCP_IMXT_ – NTCP_IMPT_) is shown in Supplementary Fig. 2, see online supplementary material. Twelve, 15, 9 and 8 patients had >10% delta NTCP for dysphagia, dysgeusia, tube-feeding dependence and xerostomia. The dose distribution of a representative case is shown in Fig. 1. Both plans were reviewed by the head and neck cancer board, and IMPT was approved as a clinical indication for all patients.

**Table 2:** DVH and NTCP comparisons for IMPT candidates

	**IMPT plan (*n* = 15)**		**IMXT plan (*n* = 15)**		***P* value**
	Median	SD	Median	SD	
Target conformity and homogeneity					
Conformity index	1.36	0.10	1.74	0.12	<0.0001^*^
Homogeneity index	1.06	0.02	1.05	0.01	0.0439^*^
Organ at risk (mean dose)					
Contralateral parotid gland	13.6	7.8	24.8	8.6	<0.0001^*^
Ipsilateral parotid gland	36.7	11.8	43.1	11.7	0.0009^*^
Oral cavity	20.9	11.0	49.6	6.9	<0.0001^*^
Pharyngeal constrictor muscle	52.9	6.8	61.1	4.8	<0.0001^*^
Superior PCM	62.5	6.4	66.6	4.0	<0.0001^*^
Middle PCM	48.0	12.7	59.4	8.5	<0.0001^*^
Inferior PCM	23.8	20.9	41.9	14.8	<0.0001^*^
Cricopharyngeal muscle	19.6	20.9	39.9	15.1	<0.0001^*^
Larynx	24.2	20.2	49.0	15.6	<0.0001^*^
Supraglottic larynx	29.8	20.6	51.3	15.5	<0.0001^*^
Glottic larynx	12.5	20.8	41.0	16.1	<0.0001^*^
NTCP (%)					
Dysphagia	19.8	12.0	37.8	12.3	<0.0001^*^
Dysgeusia	6.5	9.5	43.7	12.5	<0.0001^*^
Tube-feeding dependence	6.2	18.3	18.8	20.2	<0.0001^*^
Xerostomia	31.0	8.4	43.1	9.8	<0.0001^*^

PCM = pharyngeal constrictor muscle.

^*^Asterisks indicate statistical significance of difference at p<0.05.

### Efficacies

All patients completed the scheduled treatment. After IMPT, 13 patients completed the evaluation and had been followed-up for a median of 12.5 months at the time of analysis in June 2019. Twelve patients had a complete response. One patient experienced a partial response and had relapsed at the primary site at the 5.4 month follow-up. In the other 12 patients, 10 patients were alive with no evidence of disease, and 2 patients experienced a single distant metastasis in the follow-up period at 18.2 and 24.5 months, respectively. One patient received definitive RT to the metastasis, and there was no evidence of disease at his last follow-up. The other received chemotherapy and was waiting for radical surgery.

### Comparison of acute toxicities for the IMPT and IMXT cohorts

The median follow-up period for all 15 patients in the IMPT cohort was 7.6 months. The rates of acute toxicities in the IMPT and IMXT cohorts are summarized in Table 3. No patients experienced grade 4 or 5 toxicities. Dysphagia >grade 2 or 3 was observed in 21.4 and 7.1% of IMPT patients and in 56.6 and 33.3% of IMXT patients, respectively. Grade 2 dysgeusia was observed in 46.7% of IMPT patients and in 76.3% of IMXT patients. These toxicity rates were significantly lower in IMPT than in IMXT (all *P* < 0.05). Multivariate analysis demonstrated that IMPT was an independent factor for a lower toxicity rate for dysphagia >grade 2 (odds ratio = 0.1446, *P* = 0.0061) and dysgeusia grade 2 (odds ratio = 0.1806, *P* = 0.0173) (Table 4). To verify the effects of IMPT on dysphagia and dysgeusia, matched-pair analysis was performed. After propensity score matching, 12 pairs of IMPT and IMXT patients were selected. Dysphagia was statistically lower in IMPT than in IMXT patients (*P* < 0.05) (Table 5), but dysgeusia was not statistically different (*P* > 0.05) (Supplementary Table 4, see online supplementary material). Grade 2 or 3 xerostomia, mucositis and dermatitis in IMPT and IMXT were not significantly different, except for grade 3 dermatitis (Table 3). PEG feeding tubes were inserted into 3 (20.0%) IMPT and 55 (43.3%) IMXT patients. The median and standard deviation (SD) of body weight loss during the RT were 5.9 ± 7.3 (%) in IMPT and 5.2 ± 4.6% in IMXT, and these values were not significantly different (*P* = 0.1516).

**Table 3:** Comparison of acute-phase toxicities between IMPT and IMXT

**Endpoint**		**IMPT cohort**		**IMXT cohort**		***P* value**
		Number		Number		
Dysphagia	≥Grade 2	3/14	(21.4%)	61/108	(56.5%)	0.0115^*^
	≥Grade 3	1/14	(7.1%)	36/108	(33.3%)	0.0249^*^
Dysgeusia	≥Grade 2	7/15	(46.7%)	58/76	(76.3%)	0.0261^*^
(Grade 3 was not defined in CTCAE v4.0)						
Xerostomia	≥Grade 2	11/15	(73.3%)	49/78	(62.8%)	0.4273
	≥Grade 3	2/15	(13.3%)	10/78	(12.8%)	0.9569
Mucositis	≥Grade 2	11/15	(73.3%)	115/127	(90.6%)	0.0764
	≥Grade 3	4/15	(26.7%)	48/127	(37.8%)	0.3875
Dermatitis	≥Grade 2	10/15	(66.7%)	97/127	(76.4%)	0.4230
	≥Grade 3	4/15	(26.7%)	10/127	(7.9%)	0.0449^*^

^*^Asterisks indicate statistical significance of difference at p<0.05.

**Table 4:** Multivariate analysis of acute-phase toxicities

**Endpoint**	**Odds ratio**	**(95% CI)**	***P* value**
Dysphagia ≥ Grade 2			
IMPT vs IMXT	0.1446	0.0315– 0.6633	0.0061^*^
Age ≤ 65 vs > 65	0.4017	0.1678—0.9616	0.0364^*^
OPC vs NPC or HPC	4.4632	1.6800– 11.857	0.0015^*^
Dysphagia ≥ Grade 3			
OPC vs NPC or HPC	2.8618	1.2887– 6.3549	0.0098^*^
Dysgeusia ≥ Grade 2 (Grade 3 was not defined in CTCAE v4.0)			
IMPT vs IMXT	0.1806	0.0432– 0.7540	0.0173^*^
Age ≤ 65 vs > 65	0.2177	0.0642– 0.7383	0.0090^*^
Xerostomia ≥ Grade 2			
OPC vs NPC or HPC	5.9040	1.6510– 21.112	0.0027^*^
Xerostomia ≥ Grade 3			
(no variables correlated with the endpoint)			
Mucositis ≥ Grade 2			
Age ≤ 65 vs > 65	0.0990	0.0184– 0.5329	0.0014^*^
Mucositis ≥ Grade 3			
OPC vs NPC or HPC	3.0452	1.5003– 6.1809	0.0020^*^
Dermatitis ≥ Grade 2			
(no variables correlated with the endpoint)			
Dermatitis ≥ Grade 3			
(no variables correlated with the endpoint)			

OPC = oropharyngeal carcinoma, NPC = nasopharyngeal carcinoma, HPC = hypopharyngeal carcinoma.

^*^Asterisks indicate statistical significance of difference at p<0.05.

**Table 5:** Matched-pair analysis for dysphagia

	**All patients (pre-matching)**					**Matched patients (post-matching)**				
**Characteristics**	**IMPT cohort**		**IMXT cohort**		***P* value**	**IMPT cohort**		**IMXT cohort**		***P* value**
	*n*	%	*n*	%		*n*	%	*n*	%	
	15		127			12		12		
Sex					0.7865					1.0000
Male	14	93.3	116	91.3		11	91.7	11	91.7	
Female	1	6.7	11	8.7		1	8.3	1	8.3	
Age (years)					0.7616					1.0000
18–65	9	60.0	71	55.9		6	50.0	6	50.0	
>65	6	40.0	56	44.1		6	50.0	6	50.0	
Tumor classification^*^					0.9906					1.0000
T 0–2	9	60.0	76	59.8		7	58.3	7	58.3	
T 3–4	6	40.0	51	40.2		5	41.7	5	41.7	
Node classification^*^					0.1989					1.0000
N 0–1	6	40.0	73	57.5		6	50.0	6	50.0	
N 2–3	9	60.0	54	42.5		6	50.0	6	50.0	
Primary site					0.0073^*^					1.0000
Nasopharynx	7	46.7	15	11.8		4	33.3	4	33.3	
Oropharynx	5	33.3	58	45.7		5	41.7	5	41.7	
Hypopharynx	3	20.0	54	42.5		3	25.0	3	25.0	
Dose and fractions of radiation therapy					0.0054^*^					NA
70 Gy (E)/35 fr	13	86.7	121	95.3		12	100.0	12	100.0	
71 Gy (E)/33 fr	0	0.0	6	4.7		0	0.0	0	0.0	
71.4 Gy (E)/34 fr	2	13.3	0	0.0		0	0.0	0	0.0	
Treatment modalities					0.0724					0.2886
CCRT	10	66.7	104	81.9		10	83.3	9	75.0	
IC + CCRT	2	13.3	19	15.0		1	8.3	3	25.0	
CCRT + adjuvant Cx	3	20.0	4	3.1		1	8.3	0	0.0	
Endpoint										
Dysphagia ≥ Grade 2	3/14	21.4	61/108	56.5	0.0115^*^	3/12	25.0	9/12	75.0	0.0122^*^

^*^According to the UICC TNM-classification, 8th edition. Asterisks indicate statistical significance of difference at p<0.05.

0 Gy (E) = gray (cobalt gray equivalent), fr = fractions, Cx = chemotherapy, NA = not applicable.

### DVH analysis for the first plan on the first and second CT scans

The effects of anatomical changes on the IMPT plan with robust optimization were evaluated using the first plan on the first and second CT scans. The dose to the CTV and OARs are summarized in Supplementary Table 5, see online supplementary material. The dose to 98 (D_98%_), 95 (D_95%_), 50 (D_50%_) and 2 (D_2%_) % volume of the CTV were calculated. The D_98%_ and D_95%_ on the second CT scan were statistically lower than those on the first CT scan (both *P* < 0.0001). The median differences (delta) of D_98%_ and D_95%_ were ≥2.0%, but the deltas of D_50%_ and D_2%_ were <0.5%. The mean doses (D_mean_) of the contralateral parotid gland, oral cavity and larynx on the second CT scan were statistically higher than those on the first CT scan (*P* = 0.0172, 0.0088 and 0.0037, respectively), and the deltas were 11.3 ± 14.5, 4.9 ± 8.5 and 5.8 ± 5.7%, respectively. In 15 IMPT patients, the difference in body weight at the beginning of IMPT and at the second CT scan ranged from 0.2 to 4.0% and the median was 2.1%. The deltas of the D_mean_ of the larynx were 4.5 ± 5.5% and 9.2 ± 4.2% in the patients with body weight changes of <2.1% (*n* = 7) and ≥2.1% (*n* = 8) , respectively, and these results were statistically significant (*P* = 0.0491). The deltas for the other parameters (D_98%_, D_95%_, D_50%_ and D_2%_ of the CTV, D_max_ of the brainstem and spinal cord and D_mean_ of the parotid gland and oral cavity) were not statistically different between the two groups (all *P* > 0.05).

## DISCUSSION

This is the first report comparing the toxicities of IMPT with IMXT in patients during the same treatment period for pharyngeal cancers. The rate of physician-rated toxicity of dysphagia in the acute phase was lower in IMPT than in IMXT in the present study. This difference may have been due to the dose reduction to the pharyngeal constrictor muscle and larynx in IMPT compared to IMXT. Patients with both IMPT and IMXT were treated during the same period, and there were basically no differences in their supportive care.

Reports comparing the acute toxicity between IMPT and IMXT for pharyngeal cancer are limited. They showed better outcomes in IMPT for grade 3 acute toxicities [18], weight loss or gastrostomy-tube presence [19] and patient-reported outcomes (PROs) [20]. Manzar *et al*. recently reported that PROs, PEG-tube placement, hospitalization, narcotic requirements, acute mucositis, dysphagia and pain were better in IMPT than in volumetric modulated arc therapy (VMAT) in oropharyngeal cancer patients [21]. The rate of dysphagia was significantly lower in IMPT than in IMXT, and this result is consistent with previous reports. IMPT can contribute to lower treatment toxicity; however, this should be confirmed with other kinds of toxicity evaluations, such as PROs, and in a longer follow-up. The other potential benefit of IMPT is that treatment intensity may be intensified with IMPT. Dose-escalation or adding another concurrent chemotherapy drug may be an option in IMPT, and this should be verified in future clinical trials.

A PEG was prophylactically inserted before treatment to maintain body weight at the discretion of the attending physician at our institution. The insertion rate for IMXT was twice as high as that for IMPT, which is one reason why the rates of body weight loss were not different between IMPT and IMXT. Because the acute dysphagia rate was low in IMPT, body weight may have been maintained even if prophylactic PEG insertion was avoided.

The NTCP models used for planning comparison in the cancer board were selected based on their relative clinical importance. For example, the NTCP models of acute oral mucositis and late dysgeusia were candidates for oral toxicities. The latter was considered more important as an appropriate model for choosing a treatment method for pharyngeal cancer. We did not adopt all of the NTCP candidates because of the time and effort involved in calculating the NTCP. There were also few NTCP models for acute toxicities. As a result, the selected NTCP models were all related to late toxicities. The analysis of late and sub-acute toxicities is very important in assessing the efficacy of IMPT. Unfortunately, the short follow-up period did not allow for this analysis at the present time. Most acute toxicities are temporary and may be recovered from, which makes them relatively less important than late toxicities. Acute toxicity greatly compromises the patient’s quality of life, but not for a long period of time. This analysis primarily focused on acute toxicities, and we believe that this analysis has a significant clinical impact. However, further follow-up will allow analyses of late and sub-acute toxicities, and the results will have a more significant impact.

We reviewed published NTCP models and selected some models. Since the endpoints of these models were toxicities at 3 or 6 months after treatment [13–16], we could not verify the models using our cohort, which was evaluated in only the acute phase. After a longer follow-up period, we will evaluate subacute and late toxicities and verify the models we used.

The DVH analysis for the effects of anatomical changes revealed that the delta of D_mean_ for the larynx in the group with a greater body-weight change was statistically higher than in the group with a smaller weight change. This finding suggests that body-weight loss may easily influence anatomical changes around the neck. The delta of D_mean_ for the parotid gland and oral cavity did not appear to be related to body-weight changes. This suggests that adaptive RT should be performed regardless of body-weight change to maintain dose sparing for the parotid and oral cavity, but more research may be needed to confirm these suggestions.

In conclusion, IMPT for nasopharyngeal, oropharyngeal and hypopharyngeal cancer patients with a model-based approach may have clinical benefits for acute toxicities, especially for dysphagia. Evaluation of late toxicities and longer follow-up are needed to confirm the benefits of IMPT.

## Supplementary Material

Supplementary_material_rraa130

## Data Availability

Research data and materials are stored in an institutional repository and will be shared upon request to the corresponding author.
