# Supplementary material for: Analysis of acute-phase toxicities of intensity-modulated proton therapy using a model-based approach in pharyngeal cancer patients
Source: J Radiat Res. 2020 Dec 29;62(2):329–37. doi: 10.1093/jrr/rraa130 (PMC7948838; doi:10.1093/jrr/rraa130)
Supplement: Supplementary_material_rraa130 [file supplementary_material_rraa130.pptx]

## Slide 1
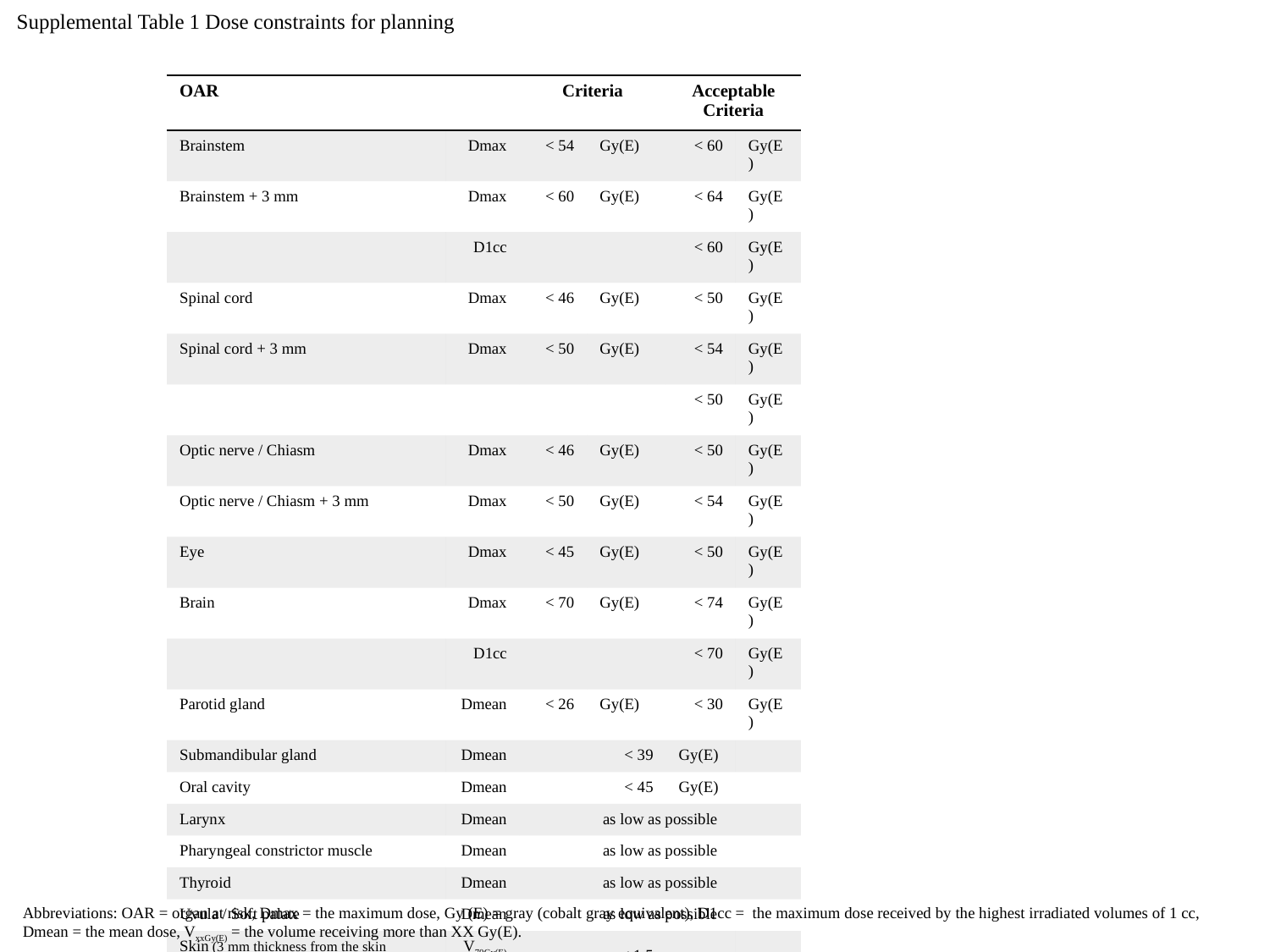

Supplemental Table 1 Dose constraints for planning
| OAR | | Criteria | | Acceptable Criteria | |
| --- | --- | --- | --- | --- | --- |
| Brainstem | Dmax | < 54 | Gy(E) | < 60 | Gy(E) |
| Brainstem + 3 mm | Dmax | < 60 | Gy(E) | < 64 | Gy(E) |
| | D1cc | | | < 60 | Gy(E) |
| Spinal cord | Dmax | < 46 | Gy(E) | < 50 | Gy(E) |
| Spinal cord + 3 mm | Dmax | < 50 | Gy(E) | < 54 | Gy(E) |
| | | | | < 50 | Gy(E) |
| Optic nerve / Chiasm | Dmax | < 46 | Gy(E) | < 50 | Gy(E) |
| Optic nerve / Chiasm + 3 mm | Dmax | < 50 | Gy(E) | < 54 | Gy(E) |
| Eye | Dmax | < 45 | Gy(E) | < 50 | Gy(E) |
| Brain | Dmax | < 70 | Gy(E) | < 74 | Gy(E) |
| | D1cc | | | < 70 | Gy(E) |
| Parotid gland | Dmean | < 26 | Gy(E) | < 30 | Gy(E) |
| Submandibular gland | Dmean | | < 39 | Gy(E) | |
| Oral cavity | Dmean | | < 45 | Gy(E) | |
| Larynx | Dmean | as low as possible | | | |
| Pharyngeal constrictor muscle | Dmean | as low as possible | | | |
| Thyroid | Dmean | as low as possible | | | |
| Uvula / Soft palate | Dmean | as low as possible | | | |
| Skin (3 mm thickness from the skin surface) | V70Gy(E) | | < 1.5 | cc | |
| | V66Gy(E) | | < 6 | cc | |
| | V60Gy(E) | | < 15 | cc | |
| | V50Gy(E) | | < 30 | cc | |
Abbreviations: OAR = organ at risk, Dmax = the maximum dose, Gy (E) = gray (cobalt gray equivalent), D1cc = the maximum dose received by the highest irradiated volumes of 1 cc, Dmean = the mean dose, VxxGy(E) = the volume receiving more than XX Gy(E).

## Slide 2
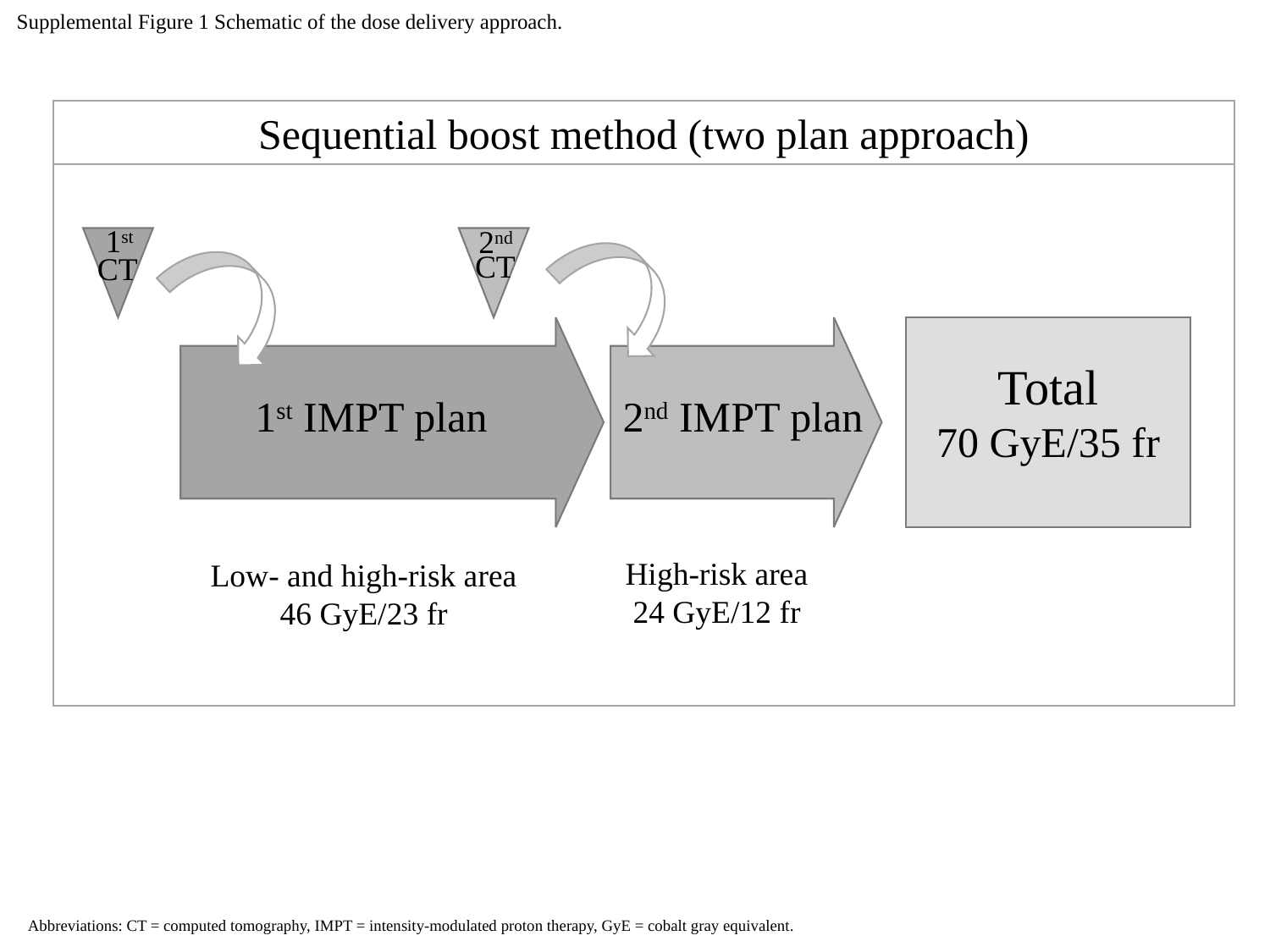

Supplemental Figure 1 Schematic of the dose delivery approach.
Sequential boost method (two plan approach)
1st
2nd
CT
CT
Total
70 GyE/35 fr
1st IMPT plan
2nd IMPT plan
High-risk area
24 GyE/12 fr
Low- and high-risk area
46 GyE/23 fr
Abbreviations: CT = computed tomography, IMPT = intensity-modulated proton therapy, GyE = cobalt gray equivalent.

## Slide 3
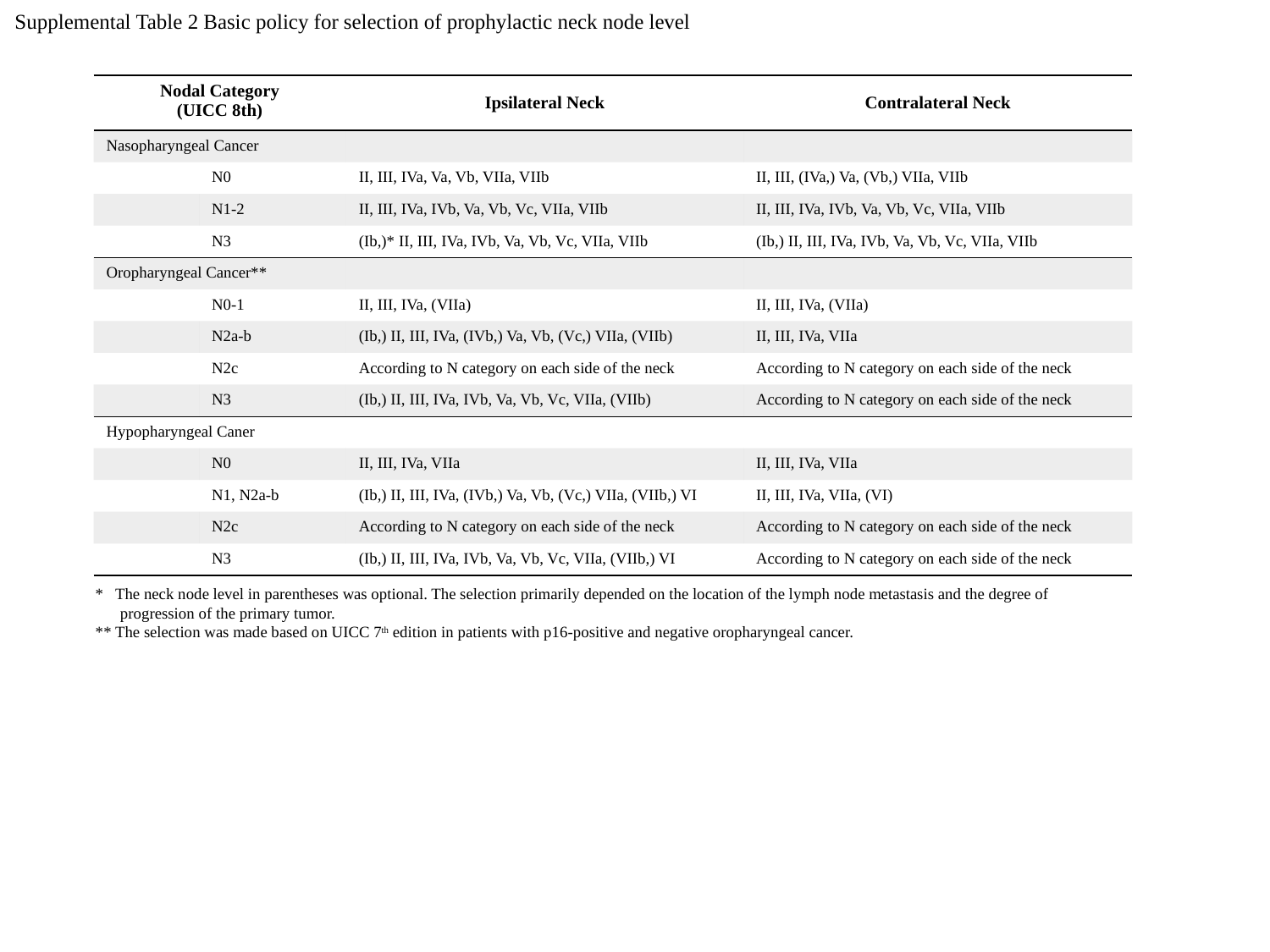

Supplemental Table 2 Basic policy for selection of prophylactic neck node level
| Nodal Category (UICC 8th) | | Ipsilateral Neck | Contralateral Neck |
| --- | --- | --- | --- |
| Nasopharyngeal Cancer | | | |
| | N0 | II, III, IVa, Va, Vb, VIIa, VIIb | II, III, (IVa,) Va, (Vb,) VIIa, VIIb |
| | N1-2 | II, III, IVa, IVb, Va, Vb, Vc, VIIa, VIIb | II, III, IVa, IVb, Va, Vb, Vc, VIIa, VIIb |
| | N3 | (Ib,)\* II, III, IVa, IVb, Va, Vb, Vc, VIIa, VIIb | (Ib,) II, III, IVa, IVb, Va, Vb, Vc, VIIa, VIIb |
| Oropharyngeal Cancer\*\* | | | |
| | N0-1 | II, III, IVa, (VIIa) | II, III, IVa, (VIIa) |
| | N2a-b | (Ib,) II, III, IVa, (IVb,) Va, Vb, (Vc,) VIIa, (VIIb) | II, III, IVa, VIIa |
| | N2c | According to N category on each side of the neck | According to N category on each side of the neck |
| | N3 | (Ib,) II, III, IVa, IVb, Va, Vb, Vc, VIIa, (VIIb) | According to N category on each side of the neck |
| Hypopharyngeal Caner | | | |
| | N0 | II, III, IVa, VIIa | II, III, IVa, VIIa |
| | N1, N2a-b | (Ib,) II, III, IVa, (IVb,) Va, Vb, (Vc,) VIIa, (VIIb,) VI | II, III, IVa, VIIa, (VI) |
| | N2c | According to N category on each side of the neck | According to N category on each side of the neck |
| | N3 | (Ib,) II, III, IVa, IVb, Va, Vb, Vc, VIIa, (VIIb,) VI | According to N category on each side of the neck |
* The neck node level in parentheses was optional. The selection primarily depended on the location of the lymph node metastasis and the degree of progression of the primary tumor.
** The selection was made based on UICC 7th edition in patients with p16-positive and negative oropharyngeal cancer.

## Slide 4
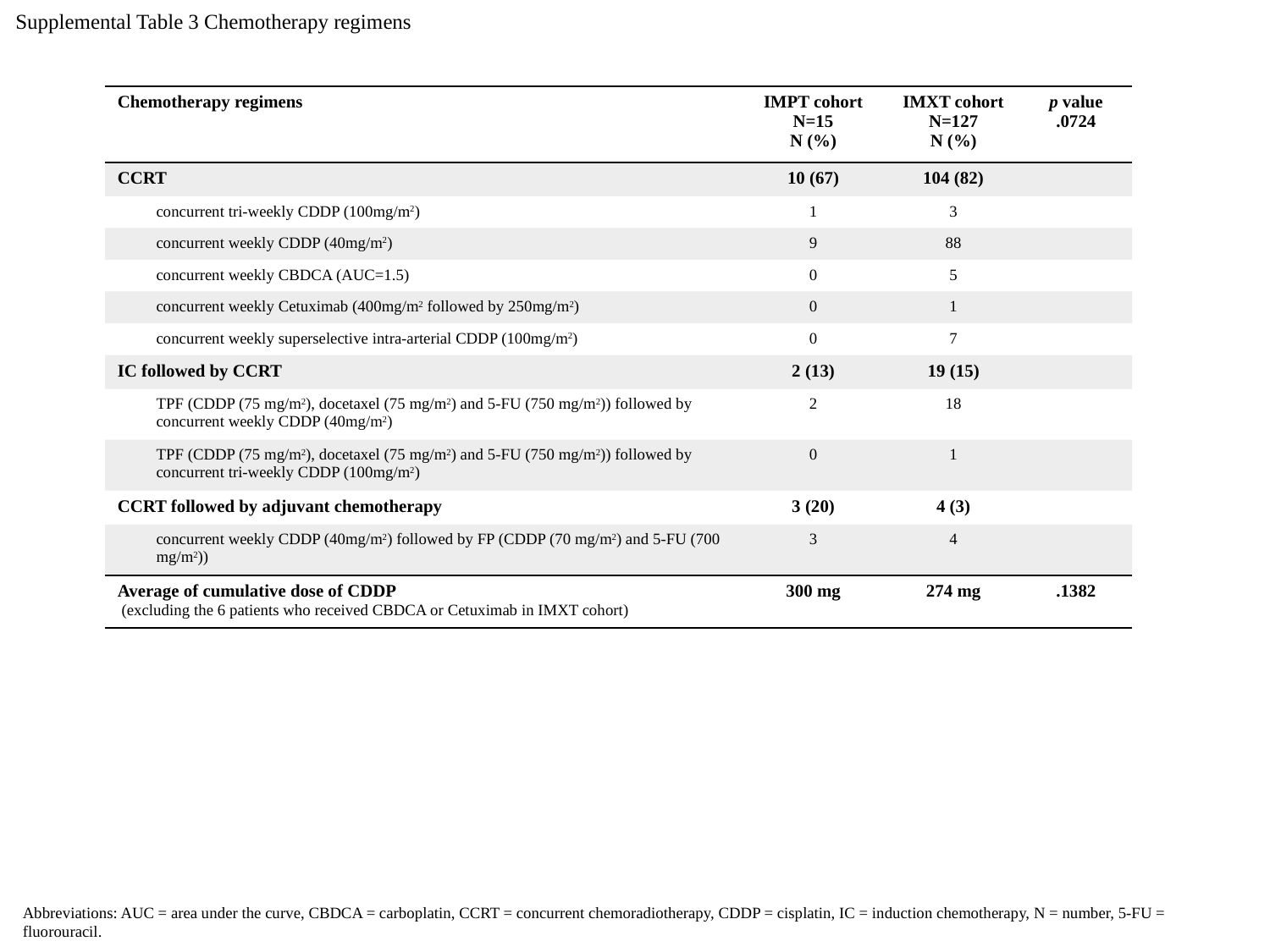

Supplemental Table 3 Chemotherapy regimens
| Chemotherapy regimens | | IMPT cohort N=15 N (%) | IMXT cohort N=127 N (%) | p value .0724 |
| --- | --- | --- | --- | --- |
| CCRT | | 10 (67) | 104 (82) | |
| | concurrent tri-weekly CDDP (100mg/m2) | 1 | 3 | |
| | concurrent weekly CDDP (40mg/m2) | 9 | 88 | |
| | concurrent weekly CBDCA (AUC=1.5) | 0 | 5 | |
| | concurrent weekly Cetuximab (400mg/m2 followed by 250mg/m2) | 0 | 1 | |
| | concurrent weekly superselective intra-arterial CDDP (100mg/m2) | 0 | 7 | |
| IC followed by CCRT | | 2 (13) | 19 (15) | |
| | TPF (CDDP (75 mg/m2), docetaxel (75 mg/m2) and 5-FU (750 mg/m2)) followed by concurrent weekly CDDP (40mg/m2) | 2 | 18 | |
| | TPF (CDDP (75 mg/m2), docetaxel (75 mg/m2) and 5-FU (750 mg/m2)) followed by concurrent tri-weekly CDDP (100mg/m2) | 0 | 1 | |
| CCRT followed by adjuvant chemotherapy | | 3 (20) | 4 (3) | |
| | concurrent weekly CDDP (40mg/m2) followed by FP (CDDP (70 mg/m2) and 5-FU (700 mg/m2)) | 3 | 4 | |
| Average of cumulative dose of CDDP (excluding the 6 patients who received CBDCA or Cetuximab in IMXT cohort) | | 300 mg | 274 mg | .1382 |
Abbreviations: AUC = area under the curve, CBDCA = carboplatin, CCRT = concurrent chemoradiotherapy, CDDP = cisplatin, IC = induction chemotherapy, N = number, 5-FU = fluorouracil.

## Slide 5
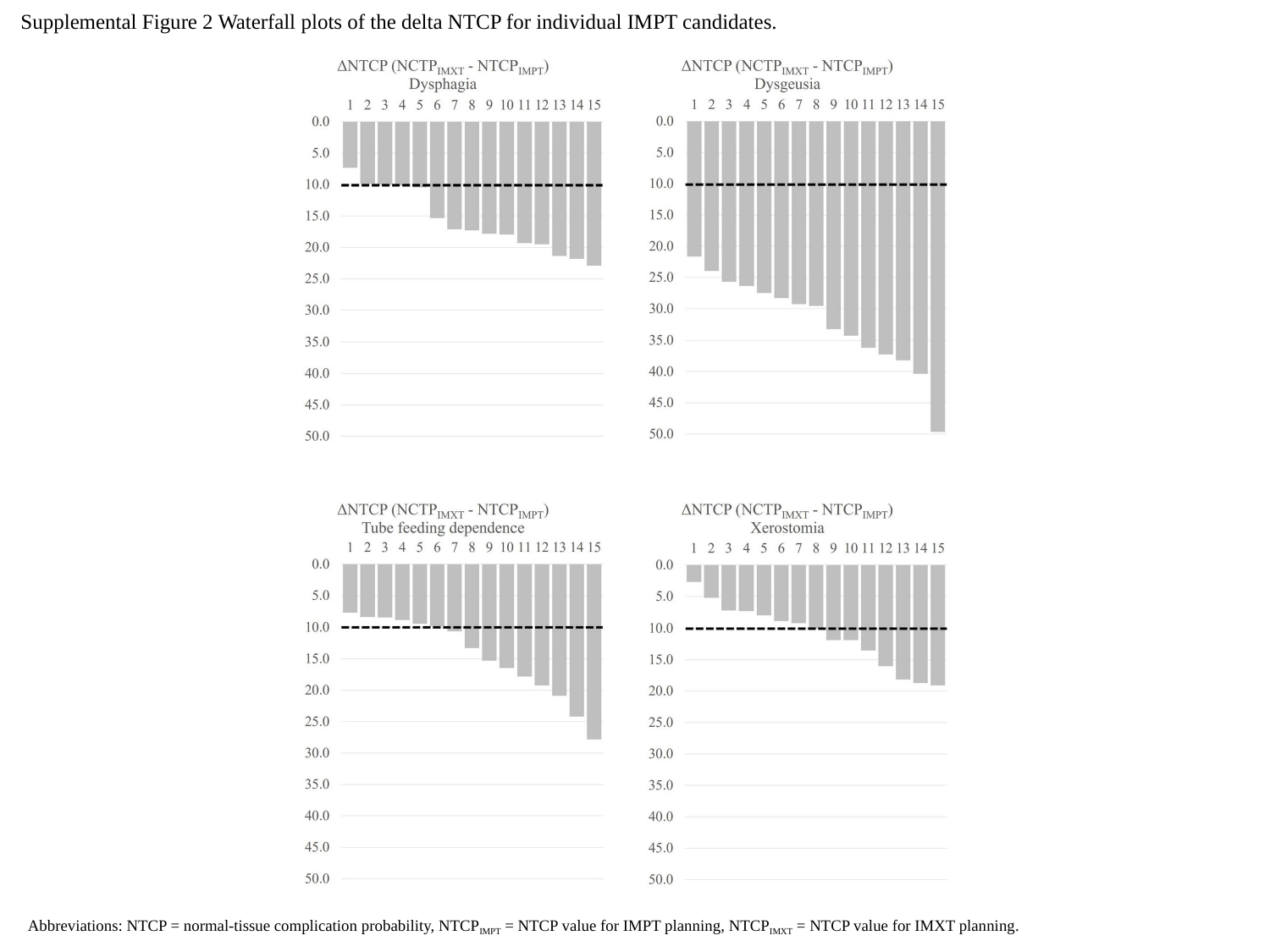

Supplemental Figure 2 Waterfall plots of the delta NTCP for individual IMPT candidates.
Abbreviations: NTCP = normal-tissue complication probability, NTCPIMPT = NTCP value for IMPT planning, NTCPIMXT = NTCP value for IMXT planning.

## Slide 6
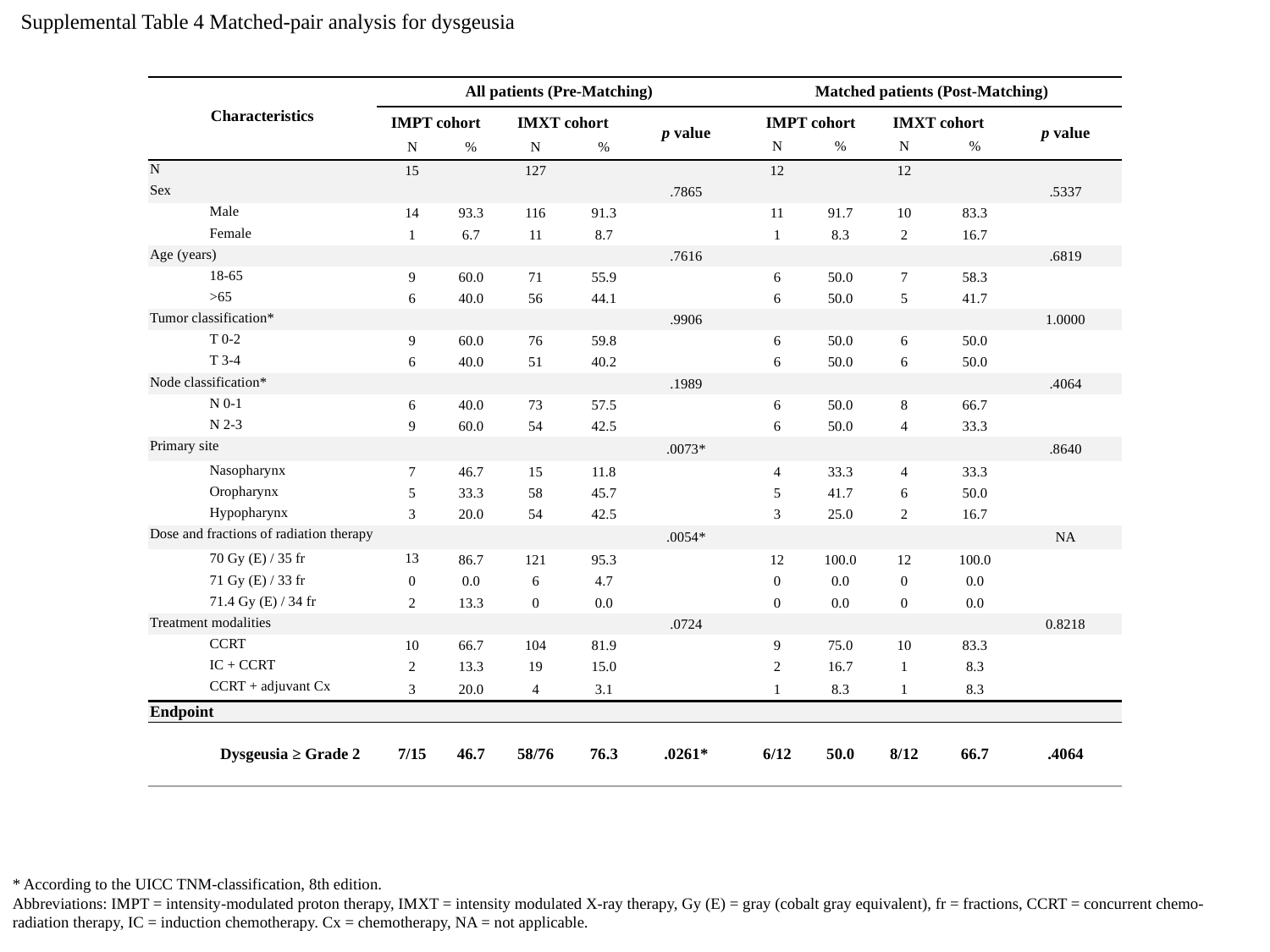

Supplemental Table 4 Matched-pair analysis for dysgeusia
| | | All patients (Pre-Matching) | | | | | Matched patients (Post-Matching) | | | | |
| --- | --- | --- | --- | --- | --- | --- | --- | --- | --- | --- | --- |
| Characteristics | | IMPT cohort | | IMXT cohort | | p value | IMPT cohort | | IMXT cohort | | p value |
| | | | | | | | N | % | N | % | |
| | | N | % | N | % | | | | | | |
| N | | 15 | | 127 | | | 12 | | 12 | | |
| Sex | | | | | | .7865 | | | | | .5337 |
| | Male | 14 | 93.3 | 116 | 91.3 | | 11 | 91.7 | 10 | 83.3 | |
| | Female | 1 | 6.7 | 11 | 8.7 | | 1 | 8.3 | 2 | 16.7 | |
| Age (years) | | | | | | .7616 | | | | | .6819 |
| | 18-65 | 9 | 60.0 | 71 | 55.9 | | 6 | 50.0 | 7 | 58.3 | |
| | >65 | 6 | 40.0 | 56 | 44.1 | | 6 | 50.0 | 5 | 41.7 | |
| Tumor classification\* | | | | | | .9906 | | | | | 1.0000 |
| | T 0-2 | 9 | 60.0 | 76 | 59.8 | | 6 | 50.0 | 6 | 50.0 | |
| | T 3-4 | 6 | 40.0 | 51 | 40.2 | | 6 | 50.0 | 6 | 50.0 | |
| Node classification\* | | | | | | .1989 | | | | | .4064 |
| | N 0-1 | 6 | 40.0 | 73 | 57.5 | | 6 | 50.0 | 8 | 66.7 | |
| | N 2-3 | 9 | 60.0 | 54 | 42.5 | | 6 | 50.0 | 4 | 33.3 | |
| Primary site | | | | | | .0073\* | | | | | .8640 |
| | Nasopharynx | 7 | 46.7 | 15 | 11.8 | | 4 | 33.3 | 4 | 33.3 | |
| | Oropharynx | 5 | 33.3 | 58 | 45.7 | | 5 | 41.7 | 6 | 50.0 | |
| | Hypopharynx | 3 | 20.0 | 54 | 42.5 | | 3 | 25.0 | 2 | 16.7 | |
| Dose and fractions of radiation therapy | | | | | | .0054\* | | | | | NA |
| | 70 Gy (E) / 35 fr | 13 | 86.7 | 121 | 95.3 | | 12 | 100.0 | 12 | 100.0 | |
| | 71 Gy (E) / 33 fr | 0 | 0.0 | 6 | 4.7 | | 0 | 0.0 | 0 | 0.0 | |
| | 71.4 Gy (E) / 34 fr | 2 | 13.3 | 0 | 0.0 | | 0 | 0.0 | 0 | 0.0 | |
| Treatment modalities | | | | | | .0724 | | | | | 0.8218 |
| | CCRT | 10 | 66.7 | 104 | 81.9 | | 9 | 75.0 | 10 | 83.3 | |
| | IC + CCRT | 2 | 13.3 | 19 | 15.0 | | 2 | 16.7 | 1 | 8.3 | |
| | CCRT + adjuvant Cx | 3 | 20.0 | 4 | 3.1 | | 1 | 8.3 | 1 | 8.3 | |
| Endpoint | | | | | | | | | | | |
| | Dysgeusia ≥ Grade 2 | 7/15 | 46.7 | 58/76 | 76.3 | .0261\* | 6/12 | 50.0 | 8/12 | 66.7 | .4064 |
* According to the UICC TNM-classification, 8th edition.
Abbreviations: IMPT = intensity-modulated proton therapy, IMXT = intensity modulated X-ray therapy, Gy (E) = gray (cobalt gray equivalent), fr = fractions, CCRT = concurrent chemo-radiation therapy, IC = induction chemotherapy. Cx = chemotherapy, NA = not applicable.

## Slide 7
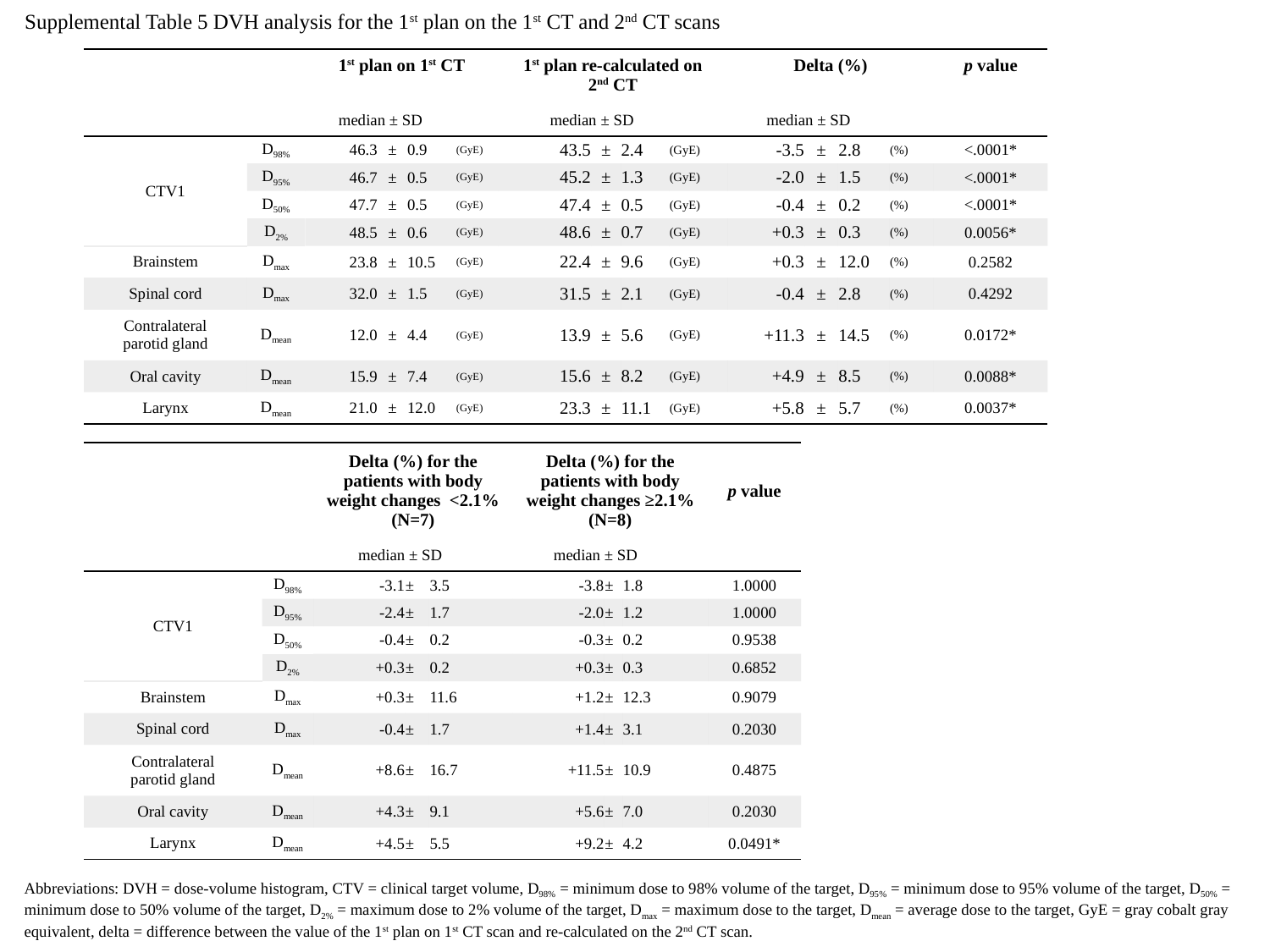

Supplemental Table 5 DVH analysis for the 1st plan on the 1st CT and 2nd CT scans
| | | 1st plan on 1st CT | | | | 1st plan re-calculated on 2nd CT | | | | Delta (%) | | | | p value |
| --- | --- | --- | --- | --- | --- | --- | --- | --- | --- | --- | --- | --- | --- | --- |
| | | median ± SD | | | | median ± SD | | | | median ± SD | | | | |
| CTV1 | D98% | 46.3 | ± | 0.9 | (GyE) | 43.5 | ± | 2.4 | (GyE) | -3.5 | ± | 2.8 | (%) | <.0001\* |
| | D95% | 46.7 | ± | 0.5 | (GyE) | 45.2 | ± | 1.3 | (GyE) | -2.0 | ± | 1.5 | (%) | <.0001\* |
| | D50% | 47.7 | ± | 0.5 | (GyE) | 47.4 | ± | 0.5 | (GyE) | -0.4 | ± | 0.2 | (%) | <.0001\* |
| | D2% | 48.5 | ± | 0.6 | (GyE) | 48.6 | ± | 0.7 | (GyE) | +0.3 | ± | 0.3 | (%) | 0.0056\* |
| Brainstem | Dmax | 23.8 | ± | 10.5 | (GyE) | 22.4 | ± | 9.6 | (GyE) | +0.3 | ± | 12.0 | (%) | 0.2582 |
| Spinal cord | Dmax | 32.0 | ± | 1.5 | (GyE) | 31.5 | ± | 2.1 | (GyE) | -0.4 | ± | 2.8 | (%) | 0.4292 |
| Contralateral parotid gland | Dmean | 12.0 | ± | 4.4 | (GyE) | 13.9 | ± | 5.6 | (GyE) | +11.3 | ± | 14.5 | (%) | 0.0172\* |
| Oral cavity | Dmean | 15.9 | ± | 7.4 | (GyE) | 15.6 | ± | 8.2 | (GyE) | +4.9 | ± | 8.5 | (%) | 0.0088\* |
| Larynx | Dmean | 21.0 | ± | 12.0 | (GyE) | 23.3 | ± | 11.1 | (GyE) | +5.8 | ± | 5.7 | (%) | 0.0037\* |
| | | Delta (%) for the patients with body weight changes <2.1% (N=7) | | | Delta (%) for the patients with body weight changes ≥2.1% (N=8) | | | p value |
| --- | --- | --- | --- | --- | --- | --- | --- | --- |
| | | median ± SD | | | median ± SD | | | |
| CTV1 | D98% | -3.1 | ± | 3.5 | -3.8 | ± | 1.8 | 1.0000 |
| | D95% | -2.4 | ± | 1.7 | -2.0 | ± | 1.2 | 1.0000 |
| | D50% | -0.4 | ± | 0.2 | -0.3 | ± | 0.2 | 0.9538 |
| | D2% | +0.3 | ± | 0.2 | +0.3 | ± | 0.3 | 0.6852 |
| Brainstem | Dmax | +0.3 | ± | 11.6 | +1.2 | ± | 12.3 | 0.9079 |
| Spinal cord | Dmax | -0.4 | ± | 1.7 | +1.4 | ± | 3.1 | 0.2030 |
| Contralateral parotid gland | Dmean | +8.6 | ± | 16.7 | +11.5 | ± | 10.9 | 0.4875 |
| Oral cavity | Dmean | +4.3 | ± | 9.1 | +5.6 | ± | 7.0 | 0.2030 |
| Larynx | Dmean | +4.5 | ± | 5.5 | +9.2 | ± | 4.2 | 0.0491\* |
Abbreviations: DVH = dose-volume histogram, CTV = clinical target volume, D98% = minimum dose to 98% volume of the target, D95% = minimum dose to 95% volume of the target, D50% = minimum dose to 50% volume of the target, D2% = maximum dose to 2% volume of the target, Dmax = maximum dose to the target, Dmean = average dose to the target, GyE = gray cobalt gray equivalent, delta = difference between the value of the 1st plan on 1st CT scan and re-calculated on the 2nd CT scan.
